# Brain DNA Methylation Age, Lifestyle Factors and Dementia in the Swedish Twin Registry

**DOI:** 10.1111/acel.70117

**Published:** 2025-06-10

**Authors:** Christopher E. McMurran, Ida K. Karlsson, Shayan Mostafaei, Yunzhang Wang, Lotte Gerritsen, Nancy L. Pedersen, Sara Hägg

**Affiliations:** ^1^ Department of Medical Epidemiology and Biostatistics Karolinska Institutet Stockholm SE Sweden; ^2^ Department of Clinical Neurosciences University of Cambridge Cambridge UK; ^3^ Department of Clinical Psychology Utrecht University Utrecht CS the Netherlands

**Keywords:** ageing, brain, cerebellum, dementia, DNA methylation, lifestyle, prefrontal cortex, registries

## Abstract

Advanced age is the most important risk factor for dementia. Measures of biological ageing such as DNA methylation age (DNAmAge) can give more information about the accumulation of age‐related molecular damage in different organs than chronological age alone. Using post‐mortem brain tissue from Swedish Twin Registry participants, we explored the relationship between lifestyle factors, dementia and DNAmAge measures from prefrontal cortex and cerebellum (*n* = 27 individuals) and paired blood samples (*n* = 20 individuals). We observed that smoking was associated with a higher DNAmAge deviation (PCBrainAge + 6.4 years in prefrontal cortex, CI [2.5, 10.3], *p* = 0.004). Conversely, a longer time spent in formal education was associated with a lower DNAmAge deviation (DNAmClock_Cortical_ − 4.8 years in prefrontal cortex, CI [−7.9, −1.8], *p* = 0.007). We found no significant differences between DNAmAge deviation of dementia cases versus controls, though among dementia cases there was a tendency towards higher DNAmClock_Cortical_ deviation in prefrontal cortex for those with a more advanced Braak stage on histopathological assessment (+ 3.4 years, CI [−0.68, 7.50], *p* = 0.13). There were no clear associations between DNAmAge from brain and blood samples collected prior to death. In summary, these data highlight the impact of smoking and education on biomarkers of brain ageing and emphasise the role for organ‐specific biomarkers of ageing.

## Graphical abstract

DNA methylation age (DNAmAge) uses molecular information to quantify biological ageing. We assessed brain‐specific DNAmAge among Swedish Twin Registry participants, revealing adverse associations with smoking and protective associations with education. Dementia outcomes were not clearly associated with brain DNAmAge. These data highlight potentially modifiable influences on biomarkers of brain ageing.

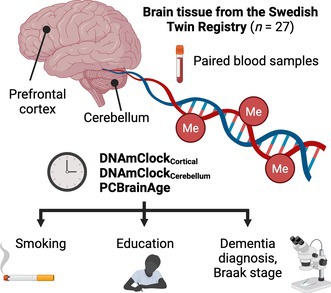

## 1 Introduction

Advanced age is the strongest risk factor for dementia (World Health Organisation 2023). The interplay between chronological age, genetics and modifiable risk factors gives rise to biological ageing: the accumulation of age‐associated biological changes such as molecular and cellular damage (Moqri et al. 2023). Biological ageing can be assessed using techniques such as DNA methylation arrays, physiological biomarkers or neuroimaging (Johnson and Shokhirev 2024), and these approaches hold promise to eventually guide clinical decision making (Herzog et al. 2024a; Livingston et al. 2024). We recently demonstrated in the Swedish Adoption/Twin Study of Aging (SATSA) that some measures of advanced blood DNA methylation age (DNAmAge) are associated with an increased future risk of dementia (McMurran et al. 2023); a phenomenon that has also been explored in other cohorts (Degerman et al. 2017; Fransquet et al. 2021; Sibbett et al. 2020; Sugden et al. 2022).

While peripheral blood is readily accessible and thus the most widely used tissue for studies of DNAmAge, there can be considerable discordance between age‐associated patterns of DNA methylation in blood and brain from the same individual (Hannon et al. 2015; Stevenson et al. 2022). Likewise, the most widely used DNA methylation clocks—which were largely validated using blood samples—are less accurate at predicting chronological age when applied to brain tissue (Shireby et al. 2020; Thrush et al. 2022). To address this, several tools have now been developed to specifically measure brain DNAmAge, using brain tissue as a training set; including DNAmClock_Cortical_ (Shireby et al. 2020), DNAmClock_Cerebellum_ (Wang et al. 2024) and PCBrainAge (Thrush et al. 2022). These tools can predict brain chronological age more accurately than blood or multi‐tissue clocks (Shireby et al. 2020; Stevenson et al. 2022; Wang et al. 2024), and there is emerging evidence they also correlate with pathological and clinical features of dementia (Grodstein et al. 2021; Thrush et al. 2022). However, their utility has so far been demonstrated in a limited number of cohorts, most of which do not include paired blood and brain samples from the same individuals. Furthermore, the relationship between brain‐specific DNAmAge and modifiable lifestyle factors (such as smoking or education) has not previously been reported. This is an important consideration as it could lead to actionable insights for healthier brain ageing.

The Swedish Twin Registry (which includes SATSA) contains detailed longitudinal data on subsets of pairs of Swedish twins with follow‐up over several decades. A number of participants (*n* = 49) donated brain tissue after their death, which has allowed us to generate measures of brain DNAmAge and ‘age deviation’, reflecting the difference between DNAmAge and chronological age at death. We studied the relationship between DNAmAge deviation of brain and blood, lifestyle factors and clinical and pathological features of dementia in this novel cohort.

## 2 Materials and Methods

### 2.1 Study Population

Post‐mortem brain samples were donated by participants of SATSA (*n* = 9 individuals) and the Study of Dementia in Swedish Twins (HARMONY, *n* = 40 individuals); two sub‐studies of the Swedish Twin Registry (Zagai et al. 2019). SATSA is a cohort study that began in 1984, based on all same‐sex twin pairs in the Swedish Twin Registry born before 1959 who were identified as reared apart matched to twin pairs reared together based on sex, date and county of birth (Finkel and Pedersen 2004). A total of 857 individuals attended at least one in‐person assessment carried out between 1985 and 2014. HARMONY is a cross‐sectional study based on a telephone interview aimed at all twin pairs aged 65 years or older carried out between 1998 and 2003 (Gatz et al. 2005). A total of 14,435 individuals participated in the interview. All twins suspected of dementia based on cognitive screening using the ‘TELE’ protocol as part of the telephone interview (Gatz et al. 1995), together with their co‐twin and a control sample of cognitively intact pairs, were invited to a clinical workup in which 1557 individuals participated.

Dementia was ascertained as part of SATSA and HARMONY, as described in detail previously (Finkel and Pedersen 2004; Gatz et al. 2005). Following an initial screening, the clinical dementia examination included physical, cognitive and neurological testing, reviews of medical records, informant interviews and laboratory tests, after which dementia diagnosis was set at multidisciplinary consensus conferences. In addition, information on dementia diagnosis after study participation was obtained from the Swedish National Patient Register and the Causes of Death Register as well as the presence of dementia medication in the Prescribed Drug Register as previously described (Karlsson et al. 2020). Register linkage was available until 31 December 2016. At this time, 294 (34.4%) participants from the SATSA in‐person assessments and 119 (7.6%) participants from the HARMONY clinical workup were alive.

Informed consent for post‐mortem brain donation was sought as part of both SATSA and HARMONY, regardless of cognitive status (Carlson et al. 2015). For both studies, once a consenting participant's relatives informed the study team about their death, their local pathology department was contacted to arrange a post‐mortem examination. Tissues were processed according to standardised methods from Brain Net Europe (Klioueva et al. 2015), with samples of prefrontal cortex and cerebellum preserved by formalin fixation paraffin embedding (FFPE). The distribution of neurofibrillary pathology in histological analysis of post‐mortem brain samples was graded 0 to VI using the method described by Braak et al. (Braak et al. 2006). Where available, blood samples collected during life from the same participants were used to calculate blood‐based DNAmAge (McMurran et al. 2023). Smoking and education data were obtained from self‐reported answers to questionnaires during follow‐up for SATSA or HARMONY. The study was approved by the Swedish Ethical Review Board/Authority (Dnr 2022‐06634, Dnr 2015/1729‐31/5).

### 2.2 Methylation Data

#### 2.2.1 Brain DNA Methylation Ages

Methylation in prefrontal cortex and cerebellar tissue was analysed with the Infinium MethylationEPIC BeadChip (Illumina Inc., San Diego, CA, USA). DNA from FFPE tissue was first extracted and bisulphite converted using the EZ DNA Methylation Gold Kit (Zymo Research Corp., Orange, CA, USA) and then hybridised to the bead chips. Quantile‐based normalisation was carried out using the *preprocessCore* package in *R*. Low‐quality samples were removed if fewer than 90% probes had a detection *p* < 0.01 and/or if sex was predicted incorrectly, leaving 27/49 prefrontal cortex samples and 26/49 cerebellar samples (*n* = 6 from SATSA; *n* = 21 from HARMONY). Brain DNA methylation array data were used to calculate the following measures of DNAmAge:

- PCHorvathAge: First‐generation multi‐tissue DNA methylation clock trained using 51 different tissue/cell types to predict chronological age. Here, we use the principal component‐based update to the original Horvath clock, a computational method to optimise its reliability (Higgins‐Chen et al. 2022; Horvath 2013).
- DNAmClock_Cortical_: Predicts chronological age, trained using cortical samples (Shireby et al. 2020).
- DNAmClock_Cerebellum_: Predicts chronological age, trained using cerebellum samples (Wang et al. 2024).
- PCBrainAge: Predicts chronological age, trained using principal components of CpG level data from dorsolateral prefrontal cortex samples (overlapping with the training set for DNAmClock_Cortical_) (Thrush et al. 2022).

DNAmClock_Cortical_ (Shireby et al. 2020), DNAmClock_Cerebellum_ (Wang et al. 2024) and PCHorvathAge (Higgins‐Chen et al. 2022; Horvath 2013) were calculated using the *dnaMethyAge* package in *R* using the clock options ‘ShirebyG_2020’, ‘CBL_specific’ and ‘PCHorvathS2013’ respectively (https://github.com/yiluyucheng/dnaMethyAge). PCBrainAge (Thrush et al. 2022) was calculated using the *CalcPCBrainAge* package in *R* (https://github.com/MorganLevineLab/calcPCBrainAge).

As these brain‐specific clocks were all developed using *Illumina* Infinium Human‐Methylation 450K BeadChip data (Higgins‐Chen et al. 2022; Shireby et al. 2020; Thrush et al. 2022; Wang et al. 2024), only data from CpG sites that are represented on the 450K array were used for the calculation of brain DNAmAge. The EPIC array includes all the CpG sites for DNAmClock_Cortical_, PCBrainAge and PCHorvathAge, and 259/275 (94.2%) of the CpG sites used for DNAmClock_Cerebellum_.

Additional publicly accessible data were downloaded from the Gene Expression Omnibus (GSE74193; (Jaffe et al. 2015)) to supplement the non‐dementia control population where specified. These 450K BeadChip data were subject to identical quality control to the Swedish Twin Registry samples, and DNAmAge was calculated in the same way. Data from adults aged 50+ free of neurological/psychiatric diagnoses were included (*n* = 102).

#### 2.2.2 Blood DNA Methylation Ages

DNA was extracted from whole blood and bisulphite converted using the EZ‐96 DNA MagPrep methylation kit for leukocytes (Zymo Research Corp., Orange, CA, USA). DNA methylation was assayed using the *Illumina* Infinium Human‐Methylation 450K BeadChip according to the manufacturer's instructions. Three blood‐based methylation age measures were generated as previously described (Higgins‐Chen et al. 2022; McMurran et al. 2023), using principal components of CpG‐level data: PCHorvathAge, PCPhenoAge and PCGrimAge.

### 2.3 Statistical Methods

DNAmAge deviation (‘AgeDev’) was calculated as a simple subtraction of chronological age from measures of DNAmAge. Age deviation was used rather than residuals (from regressing DNAmAge on chronological age) due to (1) our limited sample size to model this regression, which would risk unreliable model residuals at the extremes of chronological age and (2) to allow a more direct comparison between the different DNAmAge clocks. The association between lifestyle factors, clinical data or pathological Braak stage and brain DNAmAge deviations was assessed using linear mixed models, with DNAmAge deviation as the dependent variable and the variable of interest (e.g., dementia diagnosis, time from dementia diagnosis to death or Braak stage) used as a fixed effect, alongside chronological age, sex and neuronal cell proportion (which was calculated from methylation data using the *minfi* package; compositeCellType = ‘DLPFC’). Random intercepts were included for slide (to account for batch effect in the *Illumina* array) and for twin pair. Where specified, models were additionally adjusted for education level (compulsory only vs. more than compulsory education) and smoking status (ever vs. never). In the comparison between blood and brain from the same individual, the time between blood collection and death was also included as a fixed effect in the model. For Pearson's correlation analysis, the second member of each pair was excluded. The *p*s < 0.05 were considered statistically significant. All analyses were conducted using *R* version 4.3.1.

### 2.4 Data

The SATSA cohort has been archived through the NACDA Program on Ageing (https://www.icpsr.umich.edu/icpsrweb/ICPSR/studies/3843). Archiving for the HARMONY cohort is ongoing and data will be made available to readers on request. Brain methylation data are available in EMBL‐EBI under accession number S‐BSST2073 (https://www.ebi.ac.uk/biostudies/studies/S‐BSST2073) and blood methylation data under accession number S‐BSST1206 (https://www.ebi.ac.uk/biostudies/studies/S‐BSST1206).

## 3 Results

### 3.1 Characteristics of Brain DNAmAge Measures

DNA methylation data passing quality control criteria was available from 21 individuals diagnosed with dementia and 6 controls (Table 1). Chronological age at death was similar for those with dementia (mean age 87.3, standard deviation [SD] 6.6) and controls (86.6, SD 12.0), with dementia cases diagnosed an average of 10 years prior to death. Within the 27 individuals, there were two complete twin pairs: one monozygotic pair, one dizygotic pair. All four of these individuals had a diagnosis of dementia.

**TABLE 1:** Demographics of study population with brain methylation data available. Data are shown for those with or without a diagnosis of dementia. Only samples passing quality control criteria are shown. SD = standard deviation; IQR = interquartile range.

	Dementia cases (clinical diagnosis)	Controls
*n*	21	6
Prefrontal cortex available	21	6
Cerebellum available	20	6
Mean chronological age (SD)	87.3 (6.6)	86.6 (12.0)
Mean age of diagnosis (SD)	77.3 (8.7)	—
Male (%)	6 (28.6)	3 (50)
Ever smoker (%)	6 (28.6)	3 (50)
Beyond compulsory education (%)	8 (38.1)	2 (33.3)
Median Braak stage (IQR)	4.0 (4.0)	1.0 (1.0)
Blood methylation data available (%)	16 (76.2)	4 (66.7)
Mean age at blood sampling (SD)	80.9 (7.9)	86.6 (8.7)

**FIGURE 1:**
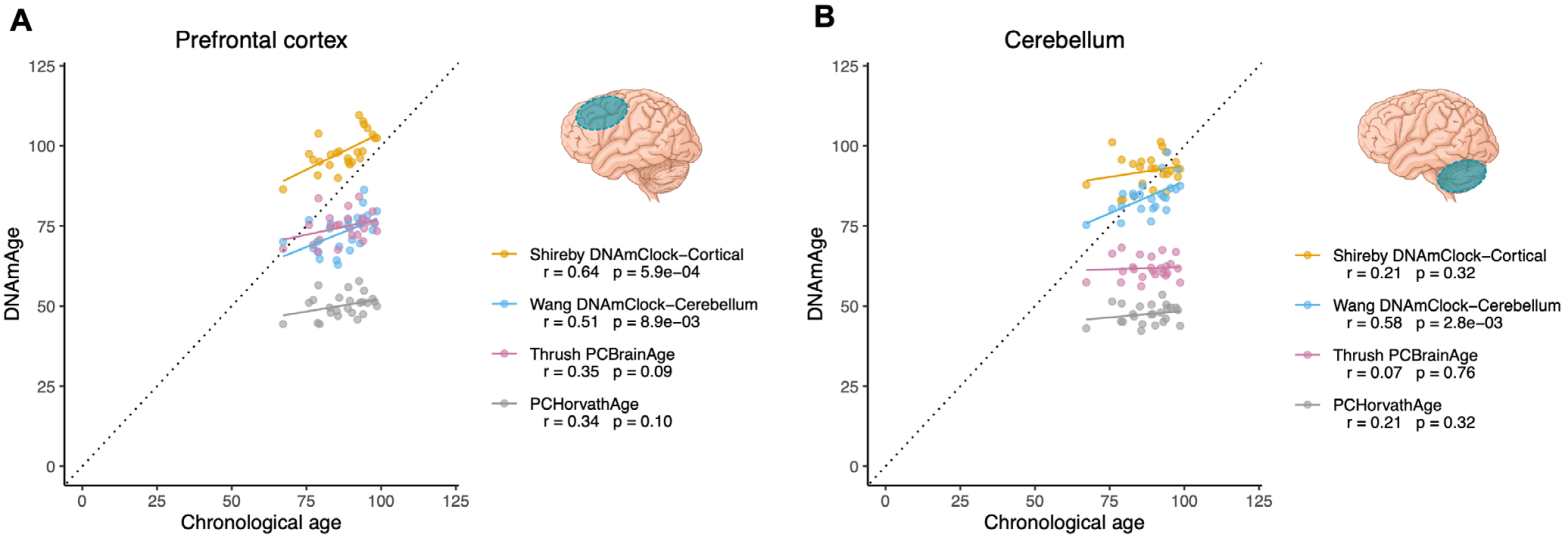
Relationships between measures of brain DNAmAge and chronological age. Linear regression of DNAmClock_Cortical_, DNAmClock_Cerebellum_, PCBrainAge and PCHorvathAge against chronological age for prefrontal cortex (A) and cerebellum (B). Pearson's correlation coefficients (*r*) and respective *p* values are shown in the legends. *n* = 25 samples for prefrontal cortex and *n* = 24 samples for cerebellum (only one member of any twin pair is represented).

Three measures of brain DNAmAge (DNAmClock_Cortical_ (Shireby et al. 2020), DNAmClock_Cerebellum_ (Wang et al. 2024) and PCBrainAge (Thrush et al. 2022) were calculated for each of these samples. For comparison, we also calculated PCHorvathAge (Higgins‐Chen et al. 2022; Horvath 2013), a multi‐tissue predictor of chronological age. In the prefrontal cortex, all DNAmAge measures correlated positively with chronological age (Figure 1A), while in the cerebellum, only the cerebellum‐specific clock (DNAmClock_Cerebellum_) correlated significantly with chronological age (*r* = 0.58, *p* = 0.003, Figure 1B). In both the prefrontal cortex and cerebellum, the brain‐specific clocks gave DNAmAge values closer to chronological age than the multi‐tissue PCHorvathAge, which was consistently 20–30 years lower than chronological age (Figure 1A,B).

DNAmAge of prefrontal cortex and cerebellum from the same individual were positively correlated for DNAmClock_Cerebellum_ (*r* = 0.42, *p* = 0.040), PCBrainAge (*r* = 0.78, *p* = 8.7 × 10^−6^) and PCHorvathAge (*r* = 0.62, *p* = 1.2 × 10^−3^), but only weakly correlated for DNAmClock_Cortical_ (*r* = 0.25, *p* = 0.25). Given these relationships with chronological age and inter‐regional correlations, and based on each clock's intended biological purpose (Shireby et al. 2020; Thrush et al. 2022; Wang et al. 2024), for subsequent analysis we focused on DNAmClock_Cortical_ and PCBrainAge in prefrontal cortex and DNAmClock_Cerebellum_ and PCBrainAge in cerebellum.

### 3.2 Lifestyle Factors and Brain DNAmAge

**FIGURE 2:**
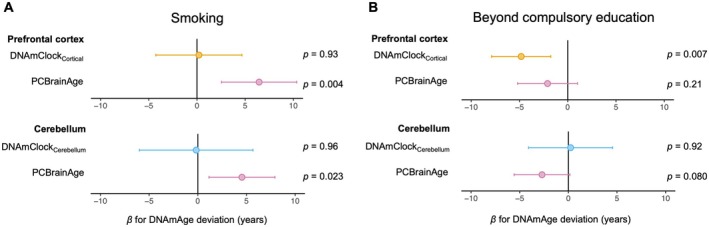
Relationships between lifestyle risk factors and brain DNAmAge deviation. Effect sizes (β) are shown for smoking (ever smoker vs. never smoker; A) and education (beyond compulsory education vs. compulsory education only; B) on deviation of different DNAmAge measures in prefrontal cortex and cerebellum. The estimated effect size and 95% confidence interval are shown, with *p* values annotated next to the plots. *n* = 27 samples for prefrontal cortex and *n* = 26 samples for cerebellum.

We next explored associations between smoking, education and brain DNAmAge. A history of smoking was associated with a higher brain DNAmAge, as measured by PCBrainAge deviation (Figure 2A). PCBrainAge deviation was on average 6 years higher in the prefrontal cortex of smokers versus never‐smokers (+6.4 years, CI [2.5, 10.3], *p* = 0.004) and 5 years higher in the cerebellum (+4.5 years, CI [1.1, 7.9], *p* = 0.023). The same association with smoking was not evident for DNAmClock_Cortical_ and DNAmClock_Cerebellum_ (Figure 2A). Using registry data on pack‐years (packs of 20 cigarettes per day × years of smoking), we assessed for evidence of a dose–response between smoking and brain DNAmAge. For all tested measures of DNAmAge, the heaviest smokers were among those with the highest DNAmAge residual (Figure S1). In adjusted linear models, there was a significant dose–response relationship between PCBrainAge and smoking pack‐years in the cerebellum (3.75 years per 10 pack‐years, CI [0.48, 7.02], *p* = 0.042; Figure S1) and a similar non‐significant trend in the prefrontal cortex (2.27 years per 10 pack‐years, CI [−1.71, 6.25], *p* = 0.28).

In contrast, continuing beyond compulsory education was associated with a lower DNAmAge deviation (Figure 2B); by 4.8 years for DNAmClock_Cortical_ and 2.1 years for PCBrainAge in the prefrontal cortex respectively (DNAmClock_Cortical_: *β* = −4.8, CI [−7.9, −1.8], *p* = 0.007; PCBrainAge: *β* = −2.1, CI [−5.2, 1.0], *p* = 0.21). In the cerebellum, a similar trend was seen for PCBrainAge (*β* = −2.7, CI [−5.6, 0.16], *p* = 0.080), but not DNAmClock_Cerebellum_ (Figure 2).

### 3.3 Association Between Brain DNAmAge and Clinicopathological Dementia Outcomes

We found no gross differences between DNAmAge deviation in dementia versus control brains in either prefrontal cortex or cerebellum (Table 2). These results were consistent when also adjusting for smoking and education as covariates (Table S1), and in sex‐stratified analysis (Table S2). In those with a diagnosis of dementia, shorter duration from dementia diagnosis to death was weakly associated with a higher PCBrainAge (Figure S2), however, this was not statistically significant (*β* for PCBrainAge in prefrontal cortex: −0.29 years/year, CI [−0.70, 0.13], *p* = 0.20).

**TABLE 2:** Comparison of DNAmAge deviation in brain tissue of dementia cases and controls. SD = standard deviation; CI = confidence interval.

	Mean DNAmAge deviation (SD)		Adjusted difference dementia vs. controls (95% CI)	*p*
	Controls	Dementia cases		
Prefrontal cortex				
*n*	6	21		
DNAmClock_Cortical_	11.9 (7.5)	10.9 (5.7)	−0.7 (−4.8, 3.3)	0.73
PCBrainAge	−12.1 (9.5)	−12.5 (7.1)	−0.9 (−4.5, 2.7)	0.63
Cerebellum				
*n*	6	20		
DNAmClock_Cerebellum_	−1.8 (8.1)	−4.6 (5.8)	−1.3 (−6.0, 3.5)	0.61
PCBrainAge	−25.4 (12.3)	−25.8 (6.9)	0.2 (−3.3, 3.8)	0.90

Given there were few brains from non‐dementia controls in our cohort (*n* = 6), we replicated the dementia versus control analysis in prefrontal cortex, combining the Swedish Twin Registry data with publicly available control data (*n* = 102 additional controls) (Jaffe et al. 2015). With this larger dataset, we again found no significant difference in DNAmAge deviation between the Swedish Twin Registry dementia cases and controls, with or without stratification by sex (Table S3).

**FIGURE 3:**
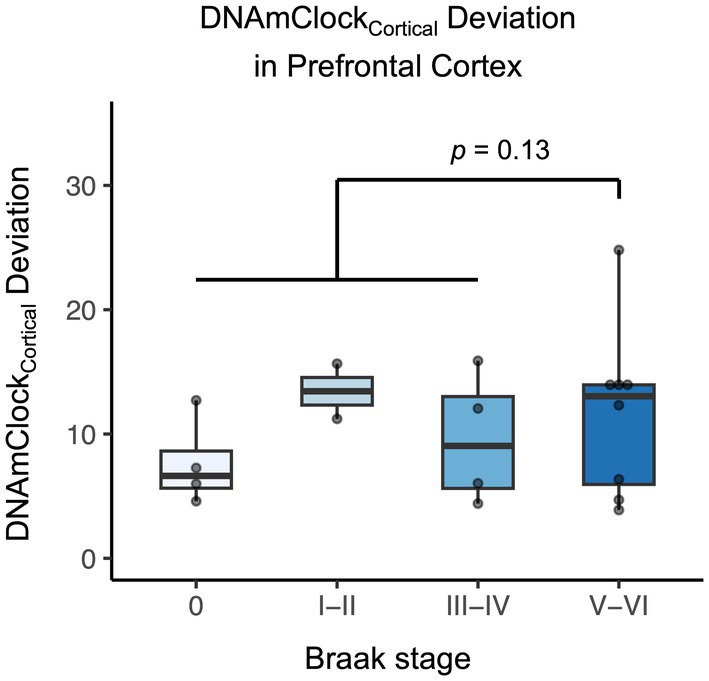
Effect of Braak stage on brain DNAmClock_Cortical_ deviation in prefrontal cortex for participants with a diagnosis of dementia. Braak stage for Alzheimer's disease is plotted on the *x*‐axis with data points and box plots displayed. The *p* value is for the adjusted difference between stages V–VI and 0–IV. *n* = 18 samples.

To investigate whether the histopathological stage of dementia is associated with DNAmAge, we grouped samples based on their Braak stage, which tracks neurofibrillary pathology across different brain regions (Figures 3 and S3). Braak staging was available for 18/21 of the individuals with a diagnosis of dementia (12 with clinically diagnosed Alzheimer's disease, 3 with clinically diagnosed vascular dementia and 3 unspecified). Considerable heterogeneity in DNAmAge deviation was observed among brain samples with neurofibrillary pathology (Braak stage > 0; Figures 3 and S3). In the prefrontal cortex, DNAmClock_Cortical_ tended to be higher in those with Braak stages V–VI (which represent tau pathology spreading to neocortical areas, such as the prefrontal cortex) compared to earlier stages, though this was not statistically significant (+ 3.4 years, CI [−0.68, 7.50], *p* = 0.13, Figure 3). The effect was reduced when also adjusting for smoking and education (+ 2.0 years, CI [−1.3, 5.3], *p* = 0.27) and was seen across both males (+ 2.5 years, CI [−0.4, 5.5], *p* = 0.34) and females (+ 4.7 years, CI [−0.9, 10.4], *p* = 0.14). There was no association between Braak stage and DNAmAge deviation in the cerebellum (Figure S3).

### 3.4 Comparison Between Blood and Brain DNAmAge

Of the 27 participants with post‐mortem brain methylation data passing quality control, 20 also had blood DNA methylation data available, from samples collected a mean of 5.0 years (SD 3.5 years) prior to death. This was at a mean chronological age of 80.9 (SD 7.9 years) for those with a dementia diagnosis (4.2 years [SD 7.5] following diagnosis) and a mean chronological age of 86.6 (SD 8.7 years) for controls. The blood methylation data were used to generate three widely used measures of blood DNAmAge: PCHorvathAge (Higgins‐Chen et al. 2022; Horvath 2013), PCPhenoAge (Higgins‐Chen et al. 2022; Levine et al. 2018) and PCGrimAge (Higgins‐Chen et al. 2022; Lu et al. 2019).

All three blood DNAmAge measures were positively correlated with chronological age (Figure S4A). However, there were no significant associations between these blood DNAmAge measures during life and any of the brain DNAmAge measures assessed post mortem (Figure S4B,C). To determine whether this finding was related to utilising different DNAmAge measures for blood and brain samples, we also compared PCHorvathAge (a multi‐tissue clock) from brain and blood of the same individuals. Again, there was no significant association between blood and brain PCHorvathAge deviation in this dataset (prefrontal cortex *β* = −0.09 years/year, CI [−0.46, 0.28], *p* = 0.64; cerebellum *β* = −0.01 years/year, CI [−0.29, 0.26], *p* = 0.93).

## 4 Discussion

In this study, we calculated measures of brain‐specific DNAmAge for post‐mortem brain samples from the Swedish Twin Registry. We confirmed previous observations that brain‐specific clocks predict chronological age more reliably than a multi‐tissue clock (Shireby et al. 2020; Thrush et al. 2022; Wang et al. 2024) and found that DNAmAge was positively correlated between prefrontal cortex and cerebellum tissue from the same individuals. Furthermore, we observed that modifiable risk factors are significantly associated with measures of brain DNAmAge: smoking exposure was associated with a dose‐dependent increase in PCBrainAge, while a higher level of education was associated with a more youthful DNAmAge measured by both DNAmClock_Cortical_ and PCBrainAge.

Smoking and education have previously been linked to similar changes in blood DNAmAge deviation (Quach et al. 2017; Si et al. 2023; Zhao et al. 2019), but this is to our knowledge the first report of these effects in brain tissue. Effects varied across the three DNAmAge measures tested—for example, in prefrontal cortex, PCBrainAge was more sensitive to smoking than DNAmClock_Cortical_. While DNAmClock_Cortical_ is derived from 347 selected CpG sites, PCBrainAge uses principal components of > 350,000 sites, so it may better reflect the widespread effects of smoking on age‐associated regions of the human methylome (Herzog et al. 2024b). We did not address the association between brain DNAmAge and other lifestyle factors in this manuscript, choosing to focus on smoking and education, which were consistently recorded in the Swedish Twin Registry sub‐studies. Other lifestyle factors, including exercise, diet, sleep, alcohol intake, socio‐demographic status and environmental pollution, can all influence biological ageing as part of the exposome (Argentieri et al. 2025) and may likewise have associations with brain DNAmAge. These lifestyle exposures tend to cluster together (Argentieri et al. 2025); for example, we suspect the protective effect of education that we observed on DNAmAge is likely to be related to a range of associated health behaviours (Liu et al. 2024).

Prior studies have found a significant increase in brain DNAmAge deviation of people with a diagnosis of dementia compared to controls, and positive associations between DNAmAge deviation and histopathological features such as tau pathology (Grodstein et al. 2021; Thrush et al. 2022). We did not find a clear difference in DNAmAge deviation between those diagnosed with dementia and controls, either with or without controlling for smoking and education. Although we were limited by the small number of control brains available in the Swedish Twin Registry, we similarly found no effect of dementia status when augmenting our controls population with external data (Jaffe et al. 2015). This may reflect heterogeneity between populations used in different studies or technical differences in data collection or tissue processing. Another consideration is that participants who reach an age to develop dementia have avoided or survived other chronic diseases earlier in life. These are in turn associated with accelerated DNAmAge measures (Assari and Pallera 2025) and will vary between cohorts. We observed a trend between advanced Braak stage and higher DNAmClock_Cortical_ deviation in the prefrontal cortex, though this effect was not statistically significant. There was no such trend in cerebellar tissue, consistent with the cerebellum being relatively spared from tau pathology in Alzheimer's disease (Larner 1997).

Finally, we did not observe any clear association between DNAmAge from blood and brain of the same 20 individuals. A caveat of our study is that different *Illumina* arrays were used to analyse brain (EPIC version 1) and blood (450K). The brain DNA methylation clocks used only CpG sites available on the 450K array, though technical differences may still influence the estimated beta values at these common sites (De Pourcq et al. 2024). It is also possible that changes occurring during the 5‐year (mean) gap between blood sampling and death might weaken any association. However, previous studies have observed a similar discordance between blood and brain methylation (Hannon et al. 2015; Stevenson et al. 2022), highlighting the need to consider organ system‐specific measures of biological ageing (Tian et al. 2023). Given that brain tissue can rarely be directly accessed in a clinical context, approaches that can evaluate brain ageing indirectly, by using molecular signatures quantified from peripheral tissues, are a promising approach for clinical translation (Oh et al. 2023; Sehgal et al. 2023).

Limitations of our study include the modest sample size and variable quality of DNA such that almost half of the samples were excluded during quality control (*n* = 27 individuals included of 49 in the initial population). Strengths include access to questionnaire data for subjects prior to death including education and smoking exposures, access to blood samples prior to death and the ability to identify dementia cases by cross‐referencing clinical ascertainment within the studies and multiple national registries to improve sensitivity and specificity (Karlsson et al. 2020). This work highlights the effect of modifiable risk factors on molecular markers of brain ageing and validates the utility of existing tissue‐specific DNA methylation clocks.

## Author Contributions

**Christopher E. McMurran** and **Sara Hägg** conceptualised and designed the project and drafted the manuscript. **Ida K. Karlsson** and **Lotte Gerritsen** carried out data collection. **Christopher E. McMurran**, **Ida K. Karlsson**, **Shayan Mostafaei**, **Yunzhang Wang** and **Sara Hägg** analysed and interpreted the data. **Nancy L. Pedersen** and **Sara Hägg** supervised the research. All authors reviewed and approved the final manuscript.

## Ethics Statement

The study was approved by the Swedish Ethical Review Board/Authority (Dnr 2022‐06634, Dnr 2015/1729‐31/5).

## Conflicts of Interest

The authors declare no conflicts of interest.

## Supporting information


Data S1.

## Data Availability

The data that support the findings of this study are openly available in BioStudies at https://www.ebi.ac.uk/biostudies/studies/S‐BSST2073 (accession number S‐BSST2073) and https://www.ebi.ac.uk/biostudies/studies/S‐BSST1206 (accession number S‐BSST1206).
