# Supplementary material for: Brain DNA Methylation Age, Lifestyle Factors and Dementia in the Swedish Twin Registry
Source: Aging Cell. 2025 Jun 10;24(8):e70117. doi: 10.1111/acel.70117 (PMC12341783; doi:10.1111/acel.70117)
Supplement: Supplementary file 1 — Data S1. [file ACEL-24-e70117-s001.pdf]

Supplementary Table 1: Comparison of DNAmAge deviation in brain tissue of dementia cases and controls after adjustment for smoking and education level. SD = standard deviation; CI = confidence interval.

	Mean DNAmAge deviation (SD)		Adjusted difference dementia vs controls (95% CI)	*p*-value
	**Controls**	**Dementia cases**		
**Prefrontal cortex**				
n	6	21		
$DNAmClock_{Cortical}$	11.9 (7.5)	10.9 (5.7)	0.3 (-3.6, 4.3)	0.87
PCBrainAge	-12.1 (9.5)	-12.5 (7.1)	1.3 (-2.3, 5.0)	0.48
**Cerebellum**				
n	6	20		
$DNAmClock_{Cerebellum}$	-1.8 (8.1)	-4.6 (5.8)	-1.3 (-6.4, 3.7)	0.61
PCBrainAge	-25.4 (12.3)	-25.8 (6.9)	0.5 (-2.6, 3.6)	0.76

Supplementary Table 2: Comparison of DNAmAge deviation in brain tissue of dementia cases and controls, stratified by sex. Sex-stratified models are not adjusted for smoking and education due to small sample size. SD = standard deviation; CI = confidence interval.

	Mean DNAmAge deviation (SD)		Adjusted difference dementia vs controls (95% CI)	*p*-value
	**Controls**	**Dementia cases**		
**Males**				
**Prefrontal cortex**				
n	3	6		
$DNAmClock_{Cortical}$	15.1 (9.3)	11.3 (4.9)	1.0 (-2.6, 4.5)	0.62
PCBrainAge	-5.4 (9.4)	-11.4 (5.6)	-1.3 (-4.6, 1.9)	0.51
**Cerebellum**				
n	3	5		
$DNAmClock_{Cerebellum}$	0.9 (9.6)	-3.1 (6.0)	-3.4 (-6.3, -0.4)	0.22
PCBrainAge	-17.8 (14.2)	-26.0 (6.6)	1.4 (0.0, 2.7)	0.26
**Females**				
**Prefrontal cortex**				
n	3	15		
$DNAmClock_{Cortical}$	8.6 (4.8)	10.8 (6.2)	-1.1 (-8.4, 6.3)	0.78
PCBrainAge	-18.9 (1.4)	-13.0 (7.7)	0.8 (-5.1, 6.7)	0.79
**Cerebellum**				
n	3	15		
$DNAmClock_{Cerebellum}$	-4.6 (7.1)	-5.1 (5.9)	-4.5 (-11.0, 2.0)	0.20
PCBrainAge	-33.1 (0.9)	-25.7 (7.2)	0.8 (-4.0, 5.5)	0.76

Supplementary Table 3: Comparison of DNAmAge deviation in prefrontal cortex tissue of dementia cases and controls with additional control data from GSE74193. Model not adjusted for smoking or education as this data was not available for the external controls. SD = standard deviation; CI = confidence interval.

	**Mean DNAmAge deviation (SD)**		**Adjusted difference dementia vs controls (95% CI)**	***p*-value**
	**Controls**	**Dementia cases**		
**Males and females**				
**Prefrontal cortex**				
n	108	21		
$DNAmClock_{Cortical}$	15.8 (3.7)	10.9 (5.7)	-0.9 (-4.1, 2.4)	0.60
PCBrainAge	-2.8 (5.2)	-12.5 (7.1)	-0.1 (-4.0, 3.8)	0.97
**Males**				
**Prefrontal cortex**				
n	67	6		
$DNAmClock_{Cortical}$	15.8 (3.8)	11.3 (4.9)	-2.2 (-6.6, 2.2)	0.34
PCBrainAge	-1.7 (4.1)	-11.4 (5.6)	-3.9 (-8.6, 0.7)	0.10
**Females**				
**Prefrontal cortex**				
n	41	15		
$DNAmClock_{Cortical}$	15.7 (3.5)	10.8 (6.2)	1.5 (-3.5, 6.6)	0.55
PCBrainAge	-4.7 (6.2)	-13.0 (7.7)	3.6 (-2.6, 9.8)	0.27

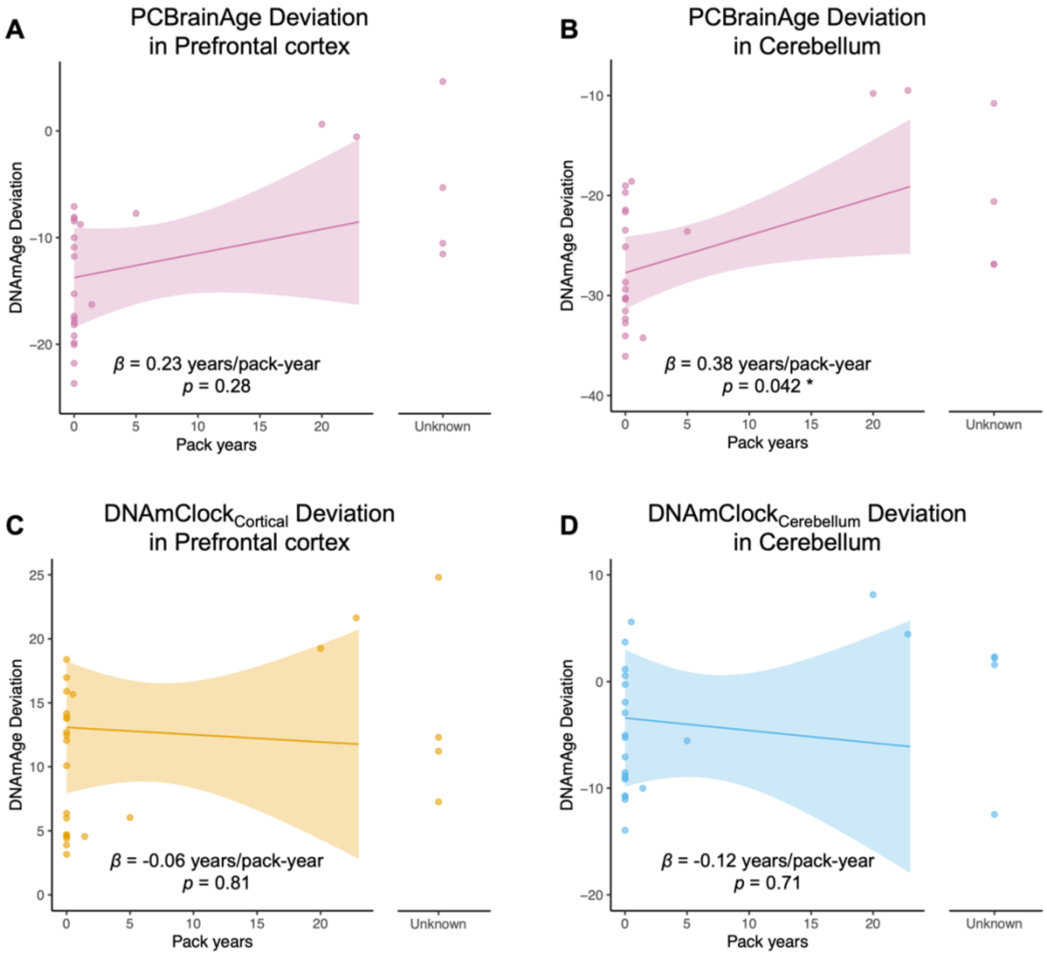

Supplementary Figure 1: Association between smoking pack-years exposure and brain DNAmAge deviation. The adjusted linear model and 95% CI are shown for PCBrainAge in prefrontal cortex (**A**) and cerebellum (**B**), for $\text{DNAmClock}_{\text{Cortical}}$ in prefrontal cortex (**C**) and $\text{DNAmClock}_{\text{Cerebellum}}$ in cerebellum (**D**). Each point represents a participant: *n* = 23 for prefrontal cortex and *n* = 22 for cerebellum. On an adjacent axis for each subfigure, the data is shown for ever-smokers with no pack-year data available (*n* = 4 for both prefrontal cortex and cerebellum).

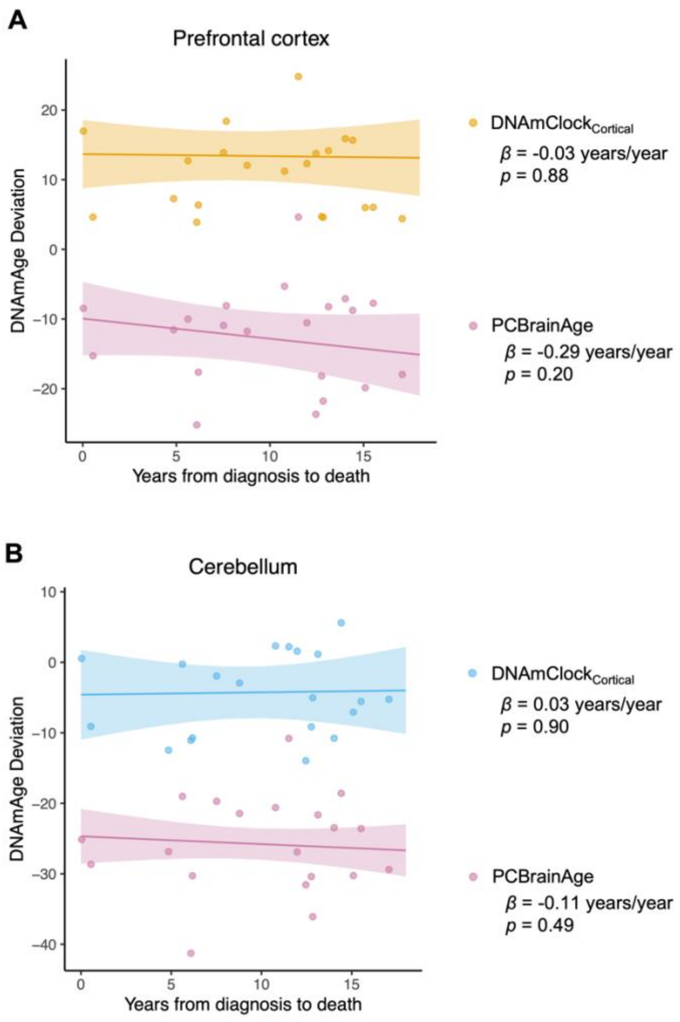

Supplementary Figure 2: Association between years from dementia diagnosis to death and brain DNAmAge deviation for dementia cases. The adjusted slope (β) and 95% CI for the models in prefrontal cortex (**A**) and cerebellum (**B**) are shown, with each point representing a participant. Models are adjusted for education and smoking status. n = 21 samples for prefrontal cortex and n = 20 samples for cerebellum.

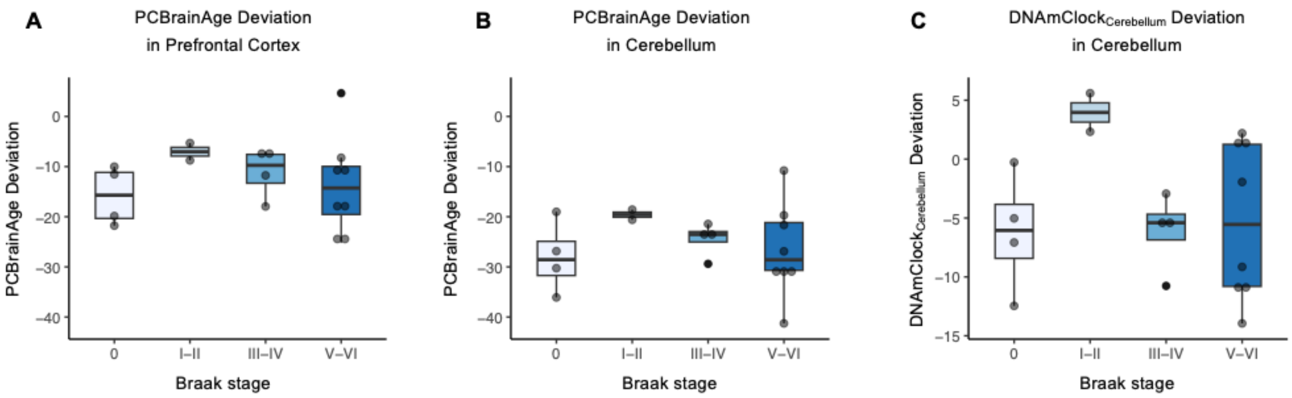

Supplementary Figure 3: Effect of Braak stage on other brain DNAmAge measure deviations in prefrontal cortex and cerebellum for participants with a diagnosis of dementia. Results are shown in prefrontal cortex using PCBrainAge (**A**) and in cerebellum using PCBrainAge (**B**) and $\text{DNAmClock}_{\text{Cerebellum}}$ (**C**). Braak stage for Alzheimer's disease is plotted on the x-axis with data points and box and whisker plots displayed. *n* = 18 samples for both prefrontal cortex and cerebellum.

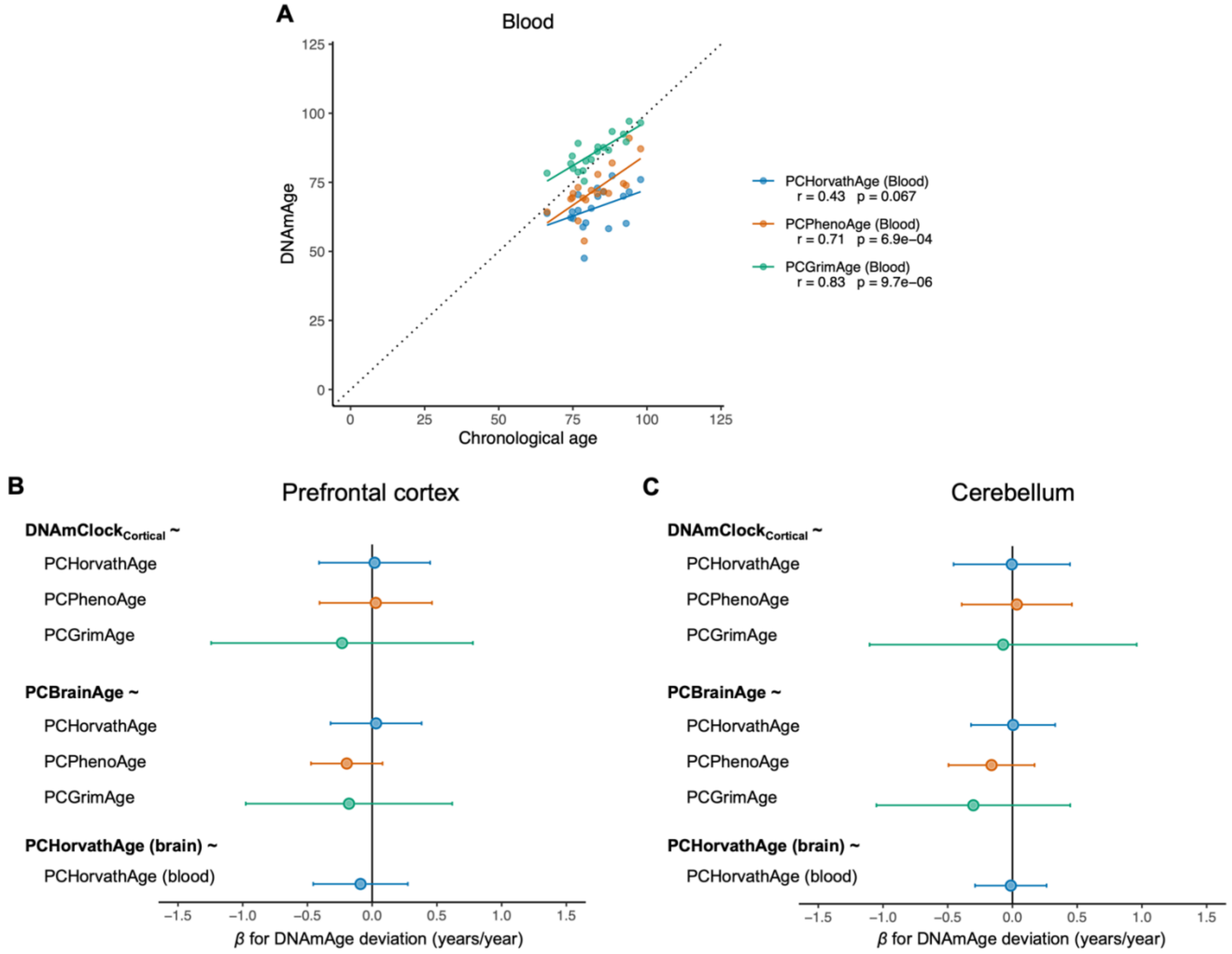

Supplementary Figure 4: Relationships between measures of blood DNAmAge, chronological age and brain DNAmAge. (**A**) Linear regression of PCHorvathAge, PCPhenoAge and PCHorvathAge against chronological age. Pearson correlation coefficients (*r*) and respective *p*-values are shown in the legends. (**B-C**) Effect sizes (β) are shown for the three different blood DNAmAge measures (non-bold) on brain DNAmAge measures (bold) in prefrontal cortex (**B**) and cerebellum (**C**). The estimated effect size and 95% confidence interval are shown. *n* = 19 blood samples in (**A**) (only one member of any twin pair is represented); *n* = 20 prefrontal cortex-blood pairs (**B**) and *n* = 19 cerebellum-blood pairs (**C**).
